# Short-Term Photovoltaic Power Prediction Method Based on Fusion of Cloud Map Features and Historical-Power Data

**DOI:** 10.3390/s26196064

**Published:** 2026-09-24

**Authors:** Wei Liu, Fangming Deng, Han Zeng, Jie Tong, Baoquan Wei

**Affiliations:** School of Electrical and Automation Engineering, East China Jiaotong University, Nanchang 330032, China; 8502@ecjtu.edu.cn (W.L.);

**Keywords:** foundation cloud map, low-rank tensor fusion, photovoltaic power prediction, cloud-image enhancement, Pyraformer network

## Abstract

Existing data-fusion-driven photovoltaic (PV) power-forecasting methods do not fully exploit cloud-image information. Simple feature concatenation cannot explicitly describe high-order interactions between cloud imagery and historical power, whereas full high-order fusion tensors introduce substantial parameter and computational overhead. This paper proposes a short-term PV power-forecasting method that fuses ground-based cloud-image features with historical-power data. MAGAN is used to enhance the brightness and texture representation of ground-based cloud images. An improved CNN-Transformer with a multi-scale separable convolution (MSC) module extracts multi-scale spatial cloud features. Low-rank tensor fusion (LTF) models cross-modal interactions between cloud-image and power features under a low-rank constraint and the fused sequence is fed into an improved Pyraformer with an i-Pyra module to forecast four 15 min steps over the next hour. Experiments using 2021 measurements from a 100 kW PV station in Jiangxi Province show that MAGAN-enhanced cloud images reach a PSNR of 7.25 dB and an SSIM of 0.75. On the independent test set, the proposed method obtains MAE, MAPE, and RMSE values of 21.8%, 17.6%, and 32.4%, respectively, reducing the three errors by 13.1%, 15.8%, and 15.2% compared with a cloud-feature and sample-weighted Pyraformer, while reducing fusion-layer parameters by 46.7% relative to full bilinear fusion.

## 1. Introduction

The uneven temporal and spatial distribution of solar energy makes PV output highly volatile and uncertain. When PV generation operates together with conventional generation, this volatility can impair power-system reliability and stability. Accurate PV power forecasting has therefore become an important research topic [1].

Conventional PV forecasting methods include physical modeling [1], persistence models [2], statistical time-series models [3,4], and gray prediction models for medium- and long-term PV generation [5,6]. These approaches can work well under stable weather and continuous power variation, but their performance is limited when PV output becomes strongly nonlinear, stochastic, and environmentally sensitive. Data-driven methods such as support vector machines [7] and artificial neural networks [8,9] improve adaptability by learning mappings between inputs and output power. Deep learning further strengthens nonlinear feature extraction, and recurrent neural networks (RNNs) [10], long short-term memory networks (LSTMs) [11,12], convolutional neural networks (CNNs) [13,14,15], as well as Transformers, have been widely applied to PV forecasting. Makris et al. [16] systematically compared the transition from statistical methods to deep learning in real operating environments and showed that deep learning is better suited to non-stationary and strongly fluctuating time series. Jannah et al. [17] reviewed recent advances and future challenges in solar power generation forecasting, highlighting the growing role of data-driven and hybrid approaches as well as continuing challenges related to data quality, meteorological uncertainty, and model generalization.

Because a single model cannot cover all influencing factors, hybrid modeling and multi-source information fusion have become major routes to higher forecasting accuracy [18]. Xiang et al. [19] proposed a TCN-ECANet-GRU hybrid model for short-term PV forecasting and reported clear gains on generation data from an Australian PV station. Cui et al. [20] combined feature construction with a TCN-Transformer model, using dilated causal convolution and self-attention to forecast PV power. Wang et al. [21] fused deep learning with temporal-correlation correction for day-ahead PV forecasting using an LSTM-RNN model. Hu et al. [22] proposed a CA-Transformer for PV output forecasting and used a Copula function to select highly correlated time-series features. Wang et al. [23] developed an LSTM-CNN hybrid model and verified its effectiveness against single models.

Most of these methods rely mainly on historical-power series and conventional meteorological variables. PV output, however, is affected by solar irradiance, ambient temperature, humidity, cloud motion, and changing weather, which together create substantial uncertainty. Keddouda et al. [24] showed that PV output depends not only on historical-power patterns but also on environmental and operating conditions, and that incorporating these variables improves accuracy. Meteorological and visual information has therefore been introduced into forecasting models. Still, conventional meteorological variables usually describe regional conditions and cannot directly represent local cloud structure, spatial distribution, or motion. Under complex weather, cloud occlusion and rapid cloud movement can cause sharp irradiance changes; relying only on historical power and regional meteorological variables is then insufficient to characterize future power trends. More recently, Zang et al. [25] proposed a sky-image-based framework for ultra-short-term PV power forecasting that explicitly considers spatial–temporal feature interaction, further demonstrating the value of jointly modeling cloud-image information and temporal dependencies.

Cloud motion and evolution are key drivers of sharp PV power fluctuations [26,27,28,29]. Ground-based cloud images provide direct visual evidence of sky conditions and have been used through cloud classification, cloud-motion analysis, and feature extraction. Hu et al. [30] predicted the drift trajectory of clouds likely to occlude the sun through cloud identification, matching, region correction, and motion prediction, extracting 13 features from ground-based images and improving forecasting accuracy by 5% over models without such features. Lu et al. [31] combined multi-scale Retinex processing with gray-level transformation to compensate illumination in cloud images. Hu et al. [32] developed an ultra-short-term PV forecasting model based on shading-cloud dynamics and RBF neural networks. Wang et al. [33] integrated ground-based cloud images with chaotic characteristics and built a two-stage cloud-speed calculation model for PV forecasting.

Despite these advances, two limitations remain. First, ground-based cloud images are easily affected by all-sky imager occlusion, fisheye distortion, and illumination variation, which reduces the reliability of image restoration and feature extraction. Second, many existing methods depend on hand-crafted features or simple concatenation, which cannot fully capture the complex relationship between spatial cloud information and temporal power information. Conventional forecasting models also have limited capacity for high-order cross-modal interaction and multi-scale temporal dependence. Improving cloud-image quality and explicitly coupling cloud spatial features with historical-power features are therefore central to short-term PV forecasting. Table 1 compares representative studies with the proposed method in terms of inputs, core methods, and research focus.

To address these issues, this paper proposes a short-term PV power-forecasting method based on low-rank tensor fusion of enhanced multi-source cloud-image features. The main contributions are as follows:

(1) A low-rank tensor fusion framework is proposed to integrate enhanced cloud-image features with historical-power data. LTF is used to model high-order interactions between spatial cloud features and temporal power features while reducing the complexity of full high-order tensor fusion.
(2) A ground-based cloud-image enhancement and multi-scale feature-extraction method is developed. Occluded regions are restored by an image-restoration network containing Swin Transformer blocks, and MAGAN improves cloud-image quality. In the improved CNN-Transformer, an MSC module strengthens extraction of cloud spatial features at different scales.
(3) An improved Pyraformer is constructed to enhance multivariate relationship modeling. The i-Pyra module strengthens interactions among fused variables, enabling the model to better learn the relationship between enhanced cloud-image features and historical-power dynamics.

## 2. Forecasting Problem Definition and Overall Framework

Ground-based cloud images describe cloud morphology and spatial distribution, whereas historical-power records describe previous PV output. Because these sources differ in observation form and temporal expression, direct combination cannot clearly define their respective roles. The proposed model centers on effective use of cloud-image information: it improves image inputs, extracts spatial features, builds multiplicative interactions between cloud images and historical power, and uses a multi-scale temporal structure to forecast future power.

### 2.1. Forecasting Problem Definition

Let t denote the forecast issue time. Ground-based cloud images and PV power are organized on a unified time index as $I_{\tau }$ and ${\;p}_{\tau }$. Let L be the input length, H the number of forecast steps, and Δt the interval between adjacent forecast indices. The target is PV power over the next H time steps, with a forecast horizon of HΔt. The task is written as follows:

(1) $${\hat{P}}_{t+1:t+H}=F_{\Theta }(I_{t-L+1:t},P_{t-L+1:t})$$

where I  and P denote the cloud-image sequence and historical-power sequence, respectively, $\hat{P}$ is the forecast power vector and $F_{\Theta }$ is the forecasting mapping determined by parameter set Θ. Only information available at or before the forecast issue time is used. In the case study, Δt = 15 min, L = 16, and H = 4. Thus, the most recent 4 h of observations are used to forecast power for the next hour. If cloud images and power are sampled at different frequencies, temporal correspondence is established before sample construction, and the same alignment rule is used for training and forecasting.

### 2.2. Overall Model Framework

Figure 1 shows the overall workflow. The cloud-image branch performs fixed-occlusion restoration, geometric distortion correction, MAGAN enhancement, and improved CNN-Transformer feature extraction. The power branch organizes historical power corresponding to the cloud-image timestamps into feature vectors. LTF then builds explicit interactions between the two feature types at each time step. The fused vectors are ordered as a temporal sequence and passed to the improved Pyraformer for multi-scale temporal modeling and future-power prediction.

## 3. Cloud-Image Enhancement and Cross-Modal Fusion Forecasting Method

### 3.1. Ground-Based Cloud-Image Preprocessing

The support arm and shading band of an all-sky imager introduce fixed occlusions, while the fisheye lens causes geometric distortion. To reduce their impact on visual representation, occluded regions are first identified by a mask and then processed through image restoration and distortion correction. Let M equal 1 in the region to be restored and 0 in valid observed regions. The restored image can be expressed as follows:

(2) $$I_{\tau }^{r}=(1-M)⊙I_{\tau }+M⊙G_{rep}(I_{\tau },M)$$

where $G_{rep}$ is the restoration generator and the circled-dot operator denotes element-wise multiplication. The expression preserves valid observed regions and fills only occluded regions with network estimates. The generator uses a UNet-style encoder–decoder containing Swin Transformer modules [34]. The encoder extracts hierarchical features, and the decoder combines skip connections to reconstruct spatial details. Local and global discriminators constrain texture within restored regions and consistency of the full image, respectively. The restoration network is an input-quality processing step, and its generated content is an estimate of missing regions. The network structure is shown in Figure 2.

A radial geometric mapping is applied to the restored fisheye cloud image. Let $\left(x_{0},y_{0}\right)$ be the image center, $\left(x_{d},y_{d}\right)$ a pixel coordinate in the distorted image, Rf the radius of the valid circular region, and rd and rc the radial distances before and after correction. Keeping the polar angle unchanged, the radial mapping is written as follows:

(3) $$r_{d}=\sqrt{{\left(x_{d}-x_{0}\right)}^{2}+{\left(y_{d}-y_{0}\right)}^{2}}.\;r_{c}=\frac{r_{d}}{\sqrt{1-{\left(r_{d}/R_{f}\right)}^{2}}}\;\theta_{d}=arctan\left(\frac{y_{d}-y_{0}}{x_{d}-x_{0}}\right)$$

The polar angle $\theta_{d}$ is determined by $arctan\left(\frac{y_{d}-y_{0}}{x_{d}-x_{0}}\right)$ while preserving quadrant information. The mapping becomes strongly magnified as $r_{d}$ approaches $R_{f}$. Therefore, the valid-sky region must be constrained and the image size unified after resampling. Geometric correction changes pixel-coordinate relationships and cannot replace restoration of occluded content.

### 3.2. Cloud-Image Enhancement and Multi-Scale Feature Extraction

#### 3.2.1. Adaptive Cloud-Image Enhancement

After restoration and geometric correction, local illumination differences can still weaken cloud-edge and texture recognition. MAGAN is therefore used for adaptive cloud-image enhancement by combining adaptive feature adjustment, mask reconstruction, and adversarial generation. The overall workflow and key modules are shown in Figure 3.

Figure 3a shows adaptive enhancement and mask reconstruction, Figure 3b shows the generative adversarial network, and Figure 3c shows the mixed-attention module in the generator.

The cloud image restored, geometrically corrected, and resized in the previous section is used as the input to the enhancement model. Its connection with preprocessing is expressed as follows:

(4) $$I_{\tau }^{e}=G_{enh}(T_{geo}(I_{\tau }^{r}))$$

where $T_{geo}$ denotes geometric correction and size normalization, $G_{enh}$ is the MAGAN enhancement mapping, and $I_{\tau }^{r}$ is the enhanced image.

As shown in Figure 3a, the adaptive enhancement module precedes mask reconstruction. It adjusts and samples input features to provide representations for subsequent encoding. During training, part of the feature map is randomly masked. The encoder extracts latent representations from visible information, and the decoder reconstructs the masked features. This reconstruction task learns relationships between visible and missing regions.

Figure 3b shows the adversarial network. The generator adopts an encoder–decoder structure and combines convolutional layers with Swin Transformer blocks for feature transformation and image reconstruction. The discriminators include local and global branches, which evaluate randomly cropped patches and the full image, respectively. The local discriminator focuses on generated-region texture and local structure, whereas the global discriminator enforces overall consistency. Adversarial learning constrains the generated image distribution and complements the feature-recovery objective of mask reconstruction.

To allow the generator to adjust feature responses according to input content, a mixed channel–spatial attention module is introduced, as shown in Figure 3c. The channel branch uses average pooling, 1 × 1 convolution, and a sigmoid function to generate channel weights, which reweight the input features. Average pooling and max pooling are then applied to the channel-weighted features, concatenated, and passed through another 1 × 1 convolution and sigmoid function to generate spatial weights. Following the connections in the figure, the original input features are finally reweighted by the spatial weights:

(5) $$F_{c}=A_{c}(F)⊙F\;F_{cs}=A_{s}(F_{c})⊙F$$

where F is the module input, $A_{c}$ and $A_{s}$ are the channel- and spatial-weight mappings, $F_{c}$ is the channel-weighted intermediate feature, and $F_{cs}$ is the module output. The channel response participates in generating the spatial weights, so the final region selection is jointly affected by channel information and spatial distribution.

#### 3.2.2. Multi-Scale Cloud-Image Feature Extraction

After adaptive enhancement, cloud edges, textures, and spatial distributions must be converted into feature representations suitable for power forecasting. Because clouds and local structures vary in scale, an improved CNN-Transformer [35] is used for visual encoding. An MSC module is introduced during feature extraction, and a SandGlass block performs channel mapping. The overall network and key modules are shown in Figure 4.

Figure 4a presents the full network and feature-extraction blocks, Figure 4b the MSC module, and Figure 4c the SandGlass module.

As shown in Figure 4a, the enhanced cloud image first passes through an input convolution block to obtain local features. Multiple feature-extraction blocks then progressively extract spatial information and transform channels. Finally, pooling aggregates spatial responses, and a fully connected layer forms the cloud-image feature vector. MSC extracts local information under different receptive fields, SandGlass further organizes channel features, and residual connections and normalization link the processing stages.

The MSC module is shown in Figure 4b. It replaces standard convolution with multi-scale separable convolution: pointwise convolution first performs cross-channel mapping, and depthwise convolutions with different kernel sizes extract local spatial information. Let F be the input feature, Z the channel-mapped feature, and $k_{j}$ x $k_{j}$ the kernel size of the j-th scale branch. Then:

(6) $$Z={PW}_{1\times 1}(F)\;Y_{j}={DW}_{k_{j}}(Z),j=1,\ldots ,J$$

The branch outputs are concatenated along the channel dimension and passed through batch normalization and an activation function to obtain the MSC output:

(7) $$F_{MSC}=GELU(BN(Concat(Y_{1},\ldots ,Y_{J})))$$

In Equation (6), PW denotes pointwise convolution, DW denotes depthwise convolution, J is the number of scale branches, Concat denotes channel-wise concatenation, and BN denotes batch normalization. The outputs of all branches must keep the same spatial size for concatenation. Pointwise convolution models inter-channel relationships, while depthwise convolution extracts spatial features within each channel. Different scale branches provide responses under different receptive fields, enabling the fused feature to contain both cloud details and broader local structures.

The MSC output then enters the SandGlass module shown in Figure 4c. This module first uses depthwise convolution to extract spatial information, then uses two convolutions for channel compression and expansion, and finally applies another depthwise convolution before passing information through a residual connection. Channel compression and expansion organize channels in a lower-dimensional intermediate representation, while depthwise convolution handles spatial information. Together with MSC, this enables multi-scale spatial responses to participate in subsequent encoding after channel transformation.

After all feature-extraction stages, pooling aggregates spatial features, and the fully connected layer maps the result to a fixed-dimensional visual vector. Denoting the visual encoder as $E_{c}$, the cloud-image feature at time tau is

(8) $$z_{\tau }^{c}=E_{c}(I_{\tau }^{e})\in R^{d_{c}}$$

where $I_{\tau }^{e}$ is the enhanced cloud image from the previous section, $z_{\tau }^{c}$ is the corresponding cloud-image feature vector, and $d_{c}$ is the feature dimension. This vector is learned by the visual encoder for the forecasting task. It represents spatial information in the cloud image and is not directly equivalent to physical quantities such as cloud thickness, transmittance, or cloud amount. It is subsequently paired with the historical-power feature at the same time index and fused through LTF.

### 3.3. Historical-Power Representation and Temporal Alignment

Historical-power records describe previous PV output and provide operating information corresponding to the cloud-image features. To associate the two observations, the power observations available up to time tau are organized as $s_{\tau }$ within the input window and mapped into a feature space by $E_{p}$:

(9) $$z_{\tau }^{p}=E_{p}(s_{\tau })\in R^{d_{p}}$$

where $z_{\tau }^{p}$ is the historical-power feature vector, $d_{p}$ is its dimension, and $E_{p}$ maps power input to the feature space. If the organized power vector is used directly for fusion, $E_{p}$ is the identity mapping. Normalization parameters for power data are determined from the training set and kept unchanged in validation, testing, and forecasting so that input scales remain consistent.

Before feature fusion, cloud-image observations and power records must be temporally aligned. The two data sources are organized on a unified time base. When sampling frequencies differ, records are paired according to sampling times, and missing or invalid matches are removed. The same alignment rule is used during training and forecasting, and only observations available at or before the forecast issue time are used to avoid future-information leakage.

After alignment, the cloud-image feature $z_{\tau }^{p}$ extracted in Section 3.2.2 and the power feature $z_{\tau }^{p}$ form a feature pair at the same time index. LTF in Section 3.4 produces the fused vector $h_{t}^{T}$. With t as the forecast issue time and L as the input length, the fused vectors are ordered chronologically to form the forecasting-network input:

(10) $$\left[\begin{array}{c} h_{t-L+1}^{T}\\ h_{t-L+2}^{T}\\ \vdots \\ h_{t}^{T} \end{array}\right]\in R^{L\times d_{h}}$$

where $h_{\tau }$ is in $R^{d_{h}}$, $d_{h}$ is the fused-feature dimension, and each row of $H_{t}$ corresponds to one time step. This organization preserves temporal order while establishing cloud-power associations: the fusion module describes interactions at the same time index, and the forecasting network learns how these interactions evolve over time.

### 3.4. Low-Rank Fusion of Cloud-Image and Power Features

After visual encoding and temporal alignment, cloud-image features and historical-power features form two inputs at the same time index. Cloud-image features describe visual spatial information, whereas historical-power features describe previous PV output. To build explicit interactions between them, LTF factorizes bilinear mapping weights and forms a cross-modal representation while controlling parameter cost. The fusion process includes feature augmentation, modality projection, element-wise interaction, and summation over rank components. The resulting fused vectors are then ordered in time and passed to the forecasting network.

#### 3.4.1. Cross-Modal Interaction Representation

Feature concatenation preserves both inputs but does not explicitly generate multiplicative terms between modalities. To model interactions between cloud-image representations and historical power, the weight-factorization idea of low-rank multimodal fusion [36] is adopted. A constant term is first appended to each feature vector:

(11) $${\tilde{z}}_{\tau }^{c}\;=\;{\left[{z_{\tau }^{c}}^{T},\;1\right]}^{T}\;{\tilde{z}}_{\tau }^{p}\;=\;{\left[{z_{\tau }^{p}}^{T},\;1\right]}^{T}$$

where $z_{\tau }^{c}$ and $z_{\tau }^{p}$ are the temporally aligned cloud-image and historical-power features, and $d_{c}$ and $d_{p}$ are their dimensions. After appending the constant terms, the outer product of the two augmented features contains cross-modal products, unimodal terms, and a constant term, allowing the fusion mapping to use both individual effects and their interactions.

Let $d_{h}$ be the fused-feature dimension. The complete bilinear mapping weight for the k-th fused output is $W_{k}$, with the corresponding matrix size. The full fusion mapping is

(12) $$h_{\tau ,k}={{\tilde{z}}_{\tau }^{c}}^{T}W_{k}{\tilde{z}}_{\tau }^{p}+b_{k},\;\;\;k=1,\;\ldots ,\;d_{h}$$

where $h_{\tau ,k}$ is the k-th component of the fused vector and $b_{k}$ is the output bias. This mapping describes second-order cross-modal interactions through pairwise products between cloud-image and power features. The weight matrices for all outputs form a third-order tensor whose dimensions correspond to cloud-image features, power features, and fused outputs.

#### 3.4.2. Fusion-Weight Factorization

A complete bilinear mapping requires a separate weight matrix for each output, and the parameter count grows with the product of the two input dimensions. To control fusion-layer cost, the weight matrix for each output is constrained to rank at most R and factorized as follows:

(13) $$W_{k}\;=\;\sum_{r=1}^{R}u_{r,k}{v_{r,k}}^{T}$$

where the terms are trainable modality-specific factors for the cloud-image and power modalities, with the indicated dimensions. The low-rank constraint acts on the cross-modal mapping weights, so each output is represented by R groups of modality factors. Substituting Equation (13) into Equation (12) gives

(14) $$h_{\tau ,k}=\sum_{r=1}^{R}\left({u_{r,k}}^{T}{\tilde{z}}_{\tau }^{c})({v_{r,k}}^{T}{\tilde{z}}_{\tau }^{p}\right)+b_{k}$$

Equation (14) shows that each rank component first projects the cloud-image and power features separately, and then uses the product of the two projections to form a cross-modal interaction. Different rank components use different projection parameters, and their sum determines the fused output. To compute all output components simultaneously, the factors for all outputs are arranged by column into matrices U and V, the fused vector can then be written as

(15) $$h_{\tau }=\sum_{r=1}^{R}\left({U_{r}}^{T}{\tilde{z}}_{\tau }^{c})\;⊙\;({V_{r}}^{T}{\tilde{z}}_{\tau }^{p}\right)+b$$

where the symbols follow the definitions above and the circled-dot operator denotes element-wise multiplication. After projection, both feature types become R-dimensional vectors; element-wise multiplication and summation over rank components directly produce the fused representation without explicitly constructing or storing the full outer-product feature for each sample.

The same fusion parameters are used at each time step and then ordered according to Equation (10) to form the fused sequence. LTF models cross-modal relationships at the same time index, and the improved Pyraformer further describes their temporal evolution.

#### 3.4.3. Parameter Cost and Expressive Constraint

When each output independently uses the factorization above and an explicit output bias is retained, the number of parameters in full bilinear fusion is

(16) $$N_{full}=d_{h}\left(d_{c}+1\right)\left(d_{p}+1\right)+d_{h}$$

With R groups of modality factors, the parameter count of the low-rank fusion layer is

(17) $$N_{LR}=Rd_{h}(d_{c}+d_{p}+2)+d_{h}$$

Therefore, the factorization reduces the fusion-layer parameter count under the condition given in Equation (17). The rank R jointly determines the expressive upper bound and computational cost of the mapping.

### 3.5. Improved Pyraformer Forecasting Model

LTF establishes interactions between cloud-image and historical-power features at the same time index, but future-power prediction also requires modeling how these fused representations change over time. Pyraformer is therefore used as the temporal backbone, and an i-Pyra module is inserted between the pyramidal-attention module and the feed-forward network to further interact and transform multi-scale temporal representations. The base model, modified structure, and i-Pyra module are shown in Figure 5a–c.

#### 3.5.1. Multi-Scale Temporal Representation

Pyraformer organizes sequence information at different temporal resolutions through intra-scale and inter-scale connections in a pyramidal structure [37]. Fine-grained nodes correspond to short-range changes, while coarse-grained nodes aggregate information over longer ranges, enabling the sequence to establish relationships across multiple time scales.

As shown in Figure 5b, the fused sequence enters the multi-scale construction module (CSCM) after input mapping and positional encoding. CSCM progressively constructs coarse-grained nodes from the input sequence to form a multi-resolution hierarchy. The pyramidal-attention module (PAM) then computes feature dependencies along intra-scale and inter-scale connections, allowing information from different temporal ranges to update the fused-sequence representation.

On this basis, the i-Pyra module is added between PAM and the feed-forward network to further process the PAM output. Following the connections in Figure 5b, the PAM output passes through residual addition and layer normalization before entering i-Pyra, and then through fully connected mapping and the feed-forward network. The outer residual connection combines the preceding representation with the transformed result to form sequence features for power prediction.

#### 3.5.2. Fixed-Window Local Interaction

The i-Pyra module is shown in Figure 5c. It contains attention computation, weight generation based on mean pooling and linear mapping, and subsequent feature mapping and concatenation. Its role is to adjust intermediate responses according to relationships among input features on top of the multi-scale representations supplied by PAM, thereby organizing sequence information for prediction.

Taking one scaled dot-product self-attention operation in the module as an example, let X in $R^{N\times d}$ be the feature matrix participating in the computation, where N is the number of nodes and d is the feature dimension. Queries, keys, and values are obtained by linear mappings:

(18) $$Q_{j}\;=\;X_{j}W_{Q}\;K_{j}\;=\;X_{j}W_{K}\;V_{j}\;=\;X_{j}W_{V}$$

where $W_{Q}$, $W_{K}$, and $W_{V}$ are trainable mapping matrices, and $d_{k}$ is the query and key dimension. Attention within a window is computed as follows:

(19) $$A_{j}\;=\;Softmax(\frac{Q_{j}{K_{j}}^{T}}{\sqrt{d_{k}}}\;+\;B_{j})V_{j}$$

where the score matrix describes matching relationships among the participating nodes. Softmax is normalized along the key-node dimension, and the resulting weights are used to aggregate value vectors. This operation enables each node output to absorb information from other nodes according to the input content.

As shown in Figure 5c, the preceding attention response is further passed through Mean, Linear, and Softmax branches to generate weights, which are used to reweight the intermediate feature. The subsequent attention and multilayer perceptron continue feature transformation, and the result is concatenated with the preceding feature to form the module output. The overall i-Pyra mapping is denoted as

(20) $$X_{out}=G_{i-Pyra}\left(X_{in};\Theta_{i}\right)$$

where $X_{in}$ is the feature representation entering i-Pyra, $X_{out}$ is the module output, and $\Theta_{i}$ is its trainable parameter set. The mapping includes the attention, weight generation, feature combination, and projection operations shown in the figure. Equations (18) and (19) describe its basic attention operation.

For non-overlapping windows of length w, the pairwise computation of single-scale window attention is approximately O($Nwd_{k}$), whereas global attention is approximately O($N^{2}d_{k}$). PAM and i-Pyra model different aspects of the sequence: PAM organizes multi-scale temporal dependence through pyramidal connections, while i-Pyra further adjusts and combines the resulting feature responses. Together they transform fused cloud-power representations into sequence features for future-power prediction.

#### 3.5.3. Power-Forecasting Output

After multi-scale modeling and i-Pyra processing, the sequence features are converted into future power forecasts by subsequent mapping and the output layer. Denoting the full forecasting network, including input mapping, CSCM, PAM, i-Pyra, and the adopted output structure, as $\hat{P}$,

(21) $${\hat{P}}_{t+1:t+H}=F_{P}(H_{t})\;\in \;R^{H}$$

where t is the forecast issue time, H is the number of forecast steps, ${\hat{P}}_{t+1:t+H}$ is the forecast vector for the next H time steps, and $H_{t}$ is the historical fused sequence organized by Equation (10). In this process, cloud-image features from visual encoding first interact with historical-power features, after which the forecasting network models the temporal dependence of the fused representation to map historical cloud images and power observations to future power.

### 3.6. Model Training Objective and Procedure

Training involves image-processing and power-forecasting objectives. In the cloud-image restoration and enhancement stage, reconstruction error and adversarial loss are used to restore occluded regions and improve image quality. In the power-forecasting stage, future measured power is the supervision signal, and mean squared error is used as the regression loss. The two stages are trained separately, and the parameters of the cloud-image feature extractor obtained in the image-processing stage are frozen during power-forecasting training.

Let $\Theta_{pred}$ be the parameter set optimized during forecasting training and $J_{pred}$ the forecasting loss. The objective is

(22) $${\Theta_{pred}}^{*}={arg\;min}_{\Theta_{pred}}\;J_{pred}(\Theta_{pred})$$

For a training set containing $N_{s}$ samples, the overall forecasting loss is

(23) $$J_{pred}=\frac{1}{N_{s}}\sum_{n=1}^{N_{s}}l_{pred}({\hat{P}}^{\left(n\right)}\;P^{\left(n\right)})$$

where $\;{\hat{P}}^{\left(n\right)}\;$ and $P^{\left(n\right)}$ are the predicted and true power vectors of the i-th sample, and ell_i is the single-sample regression loss. For multi-step forecasting, this loss measures errors over the full prediction horizon.

Training uses the Adam optimizer with an initial learning rate of $1\times 10^{-4}$, a batch size of 64, and a maximum of 10 epochs. Training stops early when validation loss does not decrease for three consecutive epochs, and the parameters with the lowest validation loss are retained. Cloud-image restoration and enhancement are trained in PyTorch (the latest stable version available at the time of the experiments), whereas power forecasting is trained in TensorFlow (the latest stable version available at the time of the experiments). The random seed is fixed at 2021. Comparative models and the proposed method use the same inputs, data split, and normalization parameters. LSTM and Transformer use four layers, a hidden dimension of 64, and four attention heads. Other settings are consistent with Table 2.

## 4. Case Study

Experiments are conducted in Python 3.9 on a computer with an NVIDIA GeForce MX150 GPU, 8 GB memory, an Intel Core i5-8250U CPU, and CUDA 11.0. Cloud-image restoration and MAGAN enhancement, including the Swin Transformer UNet structure, are implemented in PyTorch (the latest stable version available at the time of the experiments). The forecasting network and comparative models are implemented in TensorFlow (the latest stable version available at the time of the experiments). To support reproducibility, key model and training parameters are summarized in Table 2. Other settings follow the network structures described in Section 3.

### 4.1. Experimental Data

The experiments use measured operating data from a 100 kW PV station in Jiangxi Province, China, from 1 January to 31 December 2021, including ground-based cloud images, environmental monitoring data, and historical PV power. The sampling interval is 15 min, and only valid periods from 06:00 to 19:00 are retained, giving 52 sampling instants per day and 18,980 original time points with corresponding cloud images. All images are resized to 224 × 224 × 3 RGB. Missing, abnormal, or unmatched records are removed without cross-period interpolation. Cloud images and power data are matched on a unified time base using nearest-neighbor matching with a maximum allowed deviation of 7.5 min. After cleaning and synchronization, about 380 invalid groups are removed, leaving approximately 18,600 valid synchronized samples.

To avoid future-information leakage from random time-series splitting, the training, validation, and test sets are divided chronologically. Data from 1 January to 12 September 2021 are used for training, 13 September to 6 November for validation, and 7 November to 31 December for the independent test set, at an approximate ratio of 70%:15%:15%. The corresponding theoretical time points are 13,260, 2860, and 2860; after cleaning, the effective sample counts are about 12,990, 2805, and 2805. The training set is used for parameter learning, the validation set for hyperparameter and model selection, and the test set only for final evaluation. The periods do not overlap, and every test label lies within the test period. Its input window only looks backward and uses no power, cloud-image, or meteorological observations after the label time. Once hyperparameters are fixed on the validation set, the test set is used only for final performance reporting.

Table 3 reports Pearson correlation coefficients between meteorological variables and PV power to indicate information redundancy and to support invalid-record identification and typical-day selection. Absolute correlations are interpreted as follows: [0.8, 1.0] extremely strong, [0.6, 0.8) strong, [0.4, 0.6) moderate, [0.2, 0.4) weak, and [0.0, 0.2) uncorrelated. Irradiance, cloud cover, and temperature are strongly related to power; humidity is weaker, and ground wind speed and direction are weakly related. Because cloud cover can be directly represented by ground-based cloud images, meteorological variables are not included in the fusion input, avoiding redundant information and maintaining the input design in Section 3.3. They are used only for data cleaning and weather-type classification.

PV power amplitude and fluctuation differ markedly across weather types. To evaluate model behavior under different irradiance conditions, the test samples are divided into sunny, cloudy, and rainy categories for forecasting analysis.

### 4.2. Evaluation Metrics

Forecasting performance is evaluated using root mean square error (RMSE), mean absolute error (MAE), and mean absolute percentage error (MAPE). Power data are normalized according to Section 3.3. Normalization parameters are determined only from the training set and kept unchanged in validation and testing. To make errors comparable across weather conditions, MAE and RMSE are computed on the normalized power scale and reported as percentages by multiplying by 100%. MAPE is reported directly as a percentage. Because near-zero power in early morning and evening can make the MAPE denominator too small, the denominator is lower-bounded at 2% of rated power. Thus, all MAE, MAPE, and RMSE values in the results tables are reported in percent.

RMSE is calculated by Equation (24).

(24) $$RMSE=\sqrt{\frac{1}{n}\sum_{i=1}^{n}{\left({\hat{y}}_{i}-y_{i}\right)}^{2}}$$

MAE is calculated by Equation (25).

(25) $$MAE=\frac{1}{n}\sum_{i=1}^{n}\left|{\hat{y}}_{i}-y_{i}\right|$$

MAPE is calculated by Equation (26).

(26) $$MAPE=\frac{1}{N}\sum_{i=1}^{N}\left(\frac{\left|Y_{i}-{\hat{Y}}_{i}\right|}{Y_{i}}\right)\cdot 100\%$$

PSNR is calculated by Equation (27).

(27) $$PSNR=10\log_{10}\left(\frac{MAX_{I}^{2}}{MSE}\right)$$

SSIM is calculated by Equation (28).

(28) $$SSIM\left(x,y\right)=\frac{\left(2\mu_{x}\mu_{y}+C_{1}\right)\left(2\sigma_{xy}+C_{2}\right)}{\left({\mu_{X}}^{2}+{\mu_{y}}^{2}+C_{1}\right)\left(\sigma_{x}^{2}+\sigma_{y}^{2}+C_{2}\right)}$$

where the terms denote the predicted PV output power, actual PV output power, number of prediction samples, maximum possible image-pixel value, mean squared error between the evaluated and reference images, pixel values of the original and processed images, image dimensions, structural similarity index, means of images x and y, constants used for numerical stability, and standard deviations of images x and y, respectively.

### 4.3. Simulation Results

#### 4.3.1. Comparison of Ground-Based Cloud-Image Restoration Algorithms

To verify the restoration stage, masks are generated over fixed-occlusion regions caused by the all-sky imager support arm and shading band, and an additional random mask is added inside the valid observed region. Restoration results are compared with the original unobstructed image inside the masked regions. In Figure 6, from left to right are the original cloud image and the restoration results of DMFN, CTSDG, MFCL, and the proposed method; red boxes mark regions to be restored. DMFN, CTSDG, and MFCL fill most occluded regions, but structural discontinuities remain near mask boundaries and textures are less continuous than in the original image. The proposed method produces smoother transitions between masked regions and surrounding clouds and avoids obvious block-matching errors. The artificial masks still differ in shape and texture from actual support-arm occlusions, so Figure 6 is used to demonstrate reconstruction performance in missing regions.

As shown in Table 4, the proposed method achieves a PSNR of 26.852 dB, improving over DMFN, CTSDG, and MFCL by 1.528, 0.707, and 0.588 dB, respectively. Its RMSE is 11.463, lower by 2.822, 1.563, and 1.493. Table 4 metrics use the original unobstructed cloud image as the reference and are computed only in the masked regions with pixels represented on an 8 bit dynamic range.

#### 4.3.2. Comparison of Ground-Based Cloud-Image Enhancement Models

To evaluate MAGAN enhancement, it is compared with standard Retinex and bilateral-filter Retinex using the same cloud-image inputs from a TSI-880 all-sky imager dataset. The results are shown in Figure 7.

In each group of Figure 7, the images from left to right are the original cloud image, standard Retinex result, bilateral-filter Retinex result, and proposed enhancement result. The two Retinex methods provide limited gray-level improvement in cloud regions; textures remain dark, and the balance between local detail and global brightness is weaker than that of the proposed method. Quantitative results are shown in Table 5. Table 5 uses radiometrically calibrated cloud images as references and normalizes pixels to [0, 1]; therefore, PSNR values are not directly comparable with the restoration metrics in Table 4, which use an 8 bit dynamic range. SSIM is not affected by gray-level scale and can be compared across the two stages.

Table 5 shows that the proposed method reaches a PSNR of 7.25 dB and an SSIM of 0.75, improving the original image by 1.38 dB and 0.17, and the bilateral-filter Retinex by 0.28 dB and 0.06. These results indicate that MAGAN improves brightness and contrast while preserving cloud-structure information.

#### 4.3.3. Comparison of Improved Pyraformer Forecasting Models

To compare forecasting performance, three typical days are selected from the independent test set before evaluation: 24 November 2021 (sunny), 18 November 2021 (cloudy), and 24 December 2021 (rainy). These dates are not used for training or hyperparameter selection. All models use the same input length, training and validation sets, and normalization parameters. LSTM, Transformer, Pyraformer, improved Pyraformer, a cloud-feature and sample-weighted Pyraformer, and the proposed method are evaluated. Figure 8 compares predicted and actual power on the three typical days. These cases illustrate model behavior under different weather conditions; overall performance is assessed using statistics on the full independent test set.

Under sunny conditions, all models follow the overall PV power trend, but LSTM and Transformer show larger deviations near local peaks. Pyraformer-based models improve the fit, and the proposed method is closer to the measured curve at most instants, indicating that joint modeling of cloud spatial features and historical power helps under stable weather. Under cloudy conditions, PV power fluctuates more frequently, and LSTM and Transformer track abrupt changes poorly. Pyraformer and its variants capture the main trend, while the proposed method is closer to actual values during some rapid changes, showing that cloud-image information helps describe short-term fluctuations caused by cloud dynamics. Under rainy conditions, output is generally low and relative errors increase in the early morning and evening. The proposed method tracks the main trend more stably than the other models. These typical-day results are illustrative; the corresponding quantitative comparison is provided in Table 6, while robustness under low-power conditions is further evaluated using the full test-set metrics in Table 7.

For a clearer comparison, MAE, MAPE, and RMSE are calculated for each model under each weather type. The results are listed in Table 6.

Table 6 shows that the proposed method obtains average MAE, MAPE, and RMSE values of 23.6%, 19.4%, and 35.3% across the three typical days. MAE and RMSE are percentage errors on the normalized power scale. Compared with the cloud-feature and sample-weighted Pyraformer, the three metrics decrease by 11.4%, 11.0%, and 11.8%, respectively. Compared with the improved Pyraformer, they decrease by 23.3%, 29.6%, and 18.3%, compared with Pyraformer, by 22.2%, 44.0%, and 14.3%.

The three typical days reflect only selected weather conditions. Overall test-set performance is evaluated using all 2805 test samples, and the errors and daily MAE standard deviations are reported in Table 7.

As shown in Table 7, the proposed method reaches MAE, MAPE, and RMSE values of 21.8%, 17.6%, and 32.4% on the full test set. Relative to LSTM, the reductions are 36.3%, 51.8%, and 26.5%; relative to Transformer, 33.1%, 50.0%, and 24.1%; relative to Pyraformer, 24.6%, 46.8%, and 18.6%; relative to the improved Pyraformer, 20.4%, 33.1%, and 16.7%, and relative to the cloud-feature and sample-weighted Pyraformer, 13.1%, 15.8%, and 15.2%. The daily MAE standard deviation decreases from 7.8% for LSTM to 5.4% for the proposed method, indicating lower day-to-day error variability. The full test-set results are consistent with the trends observed on the typical days.

#### 4.3.4. Ablation Study

To analyze the contribution of each image-processing module in Section 3.2 to power forecasting, candidate modules are removed one at a time under identical data splits, inputs, and training settings. The adaptive enhancement module corresponds to adaptive feature adjustment in Section 3.2.1. The mask reconstruction module corresponds to the mask reconstruction process in Section 3.2.1, the mixed-attention module corresponds to Figure 3c, and the Swin Transformer module corresponds to feature transformation in the restoration network in Section 3.1 and the generator in Section 3.2.1. The ablation results are shown in Table 8. The SSIM values are computed using the reference image and scale in Table 5.

In Table 8, a check mark indicates that the module is retained, and a cross indicates that it is removed, each row differs from the full model only by the removed module. The full model achieves an SSIM of 0.75 and MAE, MAPE, and RMSE values of 23.6%, 19.4%, and 35.3%. Removing adaptive enhancement reduces SSIM to 0.67 and raises the three errors to 26.6%, 22.6%, and 39.7%, increases of 12.7%, 16.5%, and 12.5%, showing that adaptive feature adjustment provides more effective inputs for subsequent extraction. Removing mask reconstruction reduces SSIM to 0.72 and increases the errors by 7.2%, 7.7%, and 2.3%. Removing mixed attention reduces SSIM to 0.68 and increases the errors by 10.6%, 11.3%, and 11.6%. Removing Swin Transformer reduces SSIM to 0.71 and increases the errors by 8.9%, 7.7%, and 5.7%. In all cases, image-quality degradation is accompanied by higher forecasting errors, indicating that improvements in image processing transfer to forecasting performance.

#### 4.3.5. Computational Complexity Analysis

Section 3.4.3 gives the fusion-layer parameter expression, and Section 3.5.2 gives the pairwise attention computation expression. This section analytically calculates computational cost using these formulas and compares the results with full bilinear fusion and global attention. The parameter values are the same as in Table 2: input length L = 16, cloud-image feature dimension $d_{c}$ = 128, power feature dimension $d_{p}$ = 16, fused-feature dimension $d_{h}$ = 128, rank R = 8, attention key dimension $\;d_{k}$ = 32, i-Pyra window length w = 8, and number of adjacent nodes A = 3.

Table 9 shows that full bilinear fusion contains 2.81 × 10^5^ parameters. For R = 2, 4, and 8, the low-rank factorized layer contains 13.4%, 26.7%, and 53.3% as many parameters as full bilinear fusion, respectively; with R = 8, the parameter count is reduced by 46.7%. When R = 16, the parameter count reaches 106.6% of full bilinear fusion and no longer has a parameter advantage. This agrees with the condition in Equation (17); a parameter advantage exists only when $R\left(d_{c}+d_{p}+2\right)<\left(d_{c}+1\right)\left(d_{p}+1\right)$. Under the settings here, this is equivalent to R < 15.0. Therefore, R = 8 leaves a margin between expressiveness and parameter cost.

After the fused sequence enters the forecasting network, computation is mainly determined by the attention form. According to Section 3.5.2, window attention computes within windows of length w and has a single-scale pairwise cost of approximately O($Nwd_{k}$). Pyramidal attention retains only A neighboring nodes within and across scales, so its pairwise computation grows approximately linearly with sequence length. Without sparse connections, global attention has pairwise cost O($N^{2}d_{k}$). For N = 16, w = 8, and A = 3, Table 10 compares the three forms: window attention requires about half the pairwise computation of global attention, and pyramidal attention about one-fifth.

Combining Table 7, Table 9 and Table 10, the proposed method achieves MAE, MAPE, and RMSE values of 21.8%, 17.6%, and 32.4% on the independent test set. The fusion layer reduces parameters by 46.7% relative to full bilinear fusion, and i-Pyra window attention reduces pairwise computation to 50% of global attention. This comes at the cost of additional computation from cloud-image-restoration and enhancement networks. The values in Table 9 and Table 10 are analytical calculations from the formulas in Section 3.4.3 and Section 3.5.2 using the Table 2 settings; end-to-end inference latency also depends on the execution efficiency of the restoration and enhancement networks and is not measured here.

## 5. Discussion

### 5.1. Method Advantages

The proposed method improves forecasting in three linked stages. Cloud-image restoration and MAGAN enhancement improve input-image quality, and the improved CNN-Transformer extracts multi-scale spatial cloud features that provide more effective visual information. LTF explicitly models interactions between cloud spatial features and historical-power features under a low-rank constraint, reducing fusion-layer parameters by 46.7% relative to full bilinear fusion. The i-Pyra module strengthens relationship modeling among variables in the fused sequence and reduces window-attention pairwise computation to 50% of global attention. On the independent test set, MAE, MAPE, and RMSE are 21.8%, 17.6%, and 32.4%, all lower than those of LSTM, Transformer, Pyraformer, the improved Pyraformer, and the cloud-feature and sample-weighted Pyraformer. The ablation study further shows that image-quality metrics and forecasting errors change consistently.

### 5.2. Limitations and Generalizability

Several limitations remain. The data come from one PV station in 2021. Although the training, validation, and test sets are split chronologically, the test set covers only November and December, whose climatic conditions differ from those in the training period; cross-site, cross-year, and cross-climate generalization has not been verified. The restoration experiment in Figure 6 uses artificial masks, which still differ in shape and texture from real all-sky-imager support-arm occlusions. PSNR for cloud-image enhancement is computed on a normalized pixel scale and is not directly comparable with restoration-stage metrics, whereas SSIM is scale-insensitive. MAPE is amplified when power approaches zero, using 2% of rated power as the denominator lower bound affects interpretation in the early morning and evening. In addition, Table 9 and Table 10 report analytical values only. End-to-end inference latency and actual energy consumption are not measured, and simple baselines such as persistence are not included. These issues will be addressed in future work.

## 6. Conclusions

To improve the use of ground-based cloud-image features, this paper proposes a short-term PV power-forecasting method that fuses cloud-image features with historical-power data. The main conclusions are as follows:

(1) LTF explicitly models interactions between cloud-image and historical-power features under a low-rank constraint, and the improved Pyraformer uses relationships among variables for prediction. On the independent test set, the proposed method obtains MAE, MAPE, and RMSE values of 21.8%, 17.6%, and 32.4%. With R = 8, the fusion layer contains 1.50 × 10^5^ parameters, 46.7% fewer than full bilinear fusion.
(2) MAGAN, which combines Swin Transformer and generative adversarial learning, is used for ground-based cloud-image restoration and enhancement. It raises cloud-image PSNR and SSIM to 7.25 dB and 0.75, respectively. Ablation experiments show that removing adaptive enhancement, mask reconstruction, mixed attention, or Swin Transformer decreases cloud-image SSIM and increases all three forecasting errors, indicating that image-quality improvements transfer to power-forecasting performance.

These results show that combining cloud-image visual information with historical-power data while controlling fusion-layer parameter cost improves short-term PV power-forecasting accuracy. Applicability across stations and years should be verified with broader datasets.

## Figures and Tables

**Figure 1:**
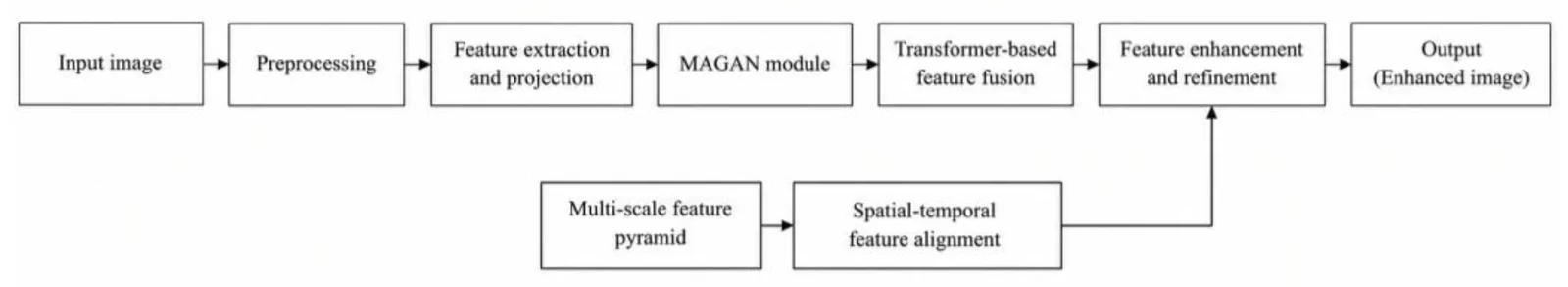
Overall framework of cloud-image and historical-power fusion forecasting.

**Figure 2:**
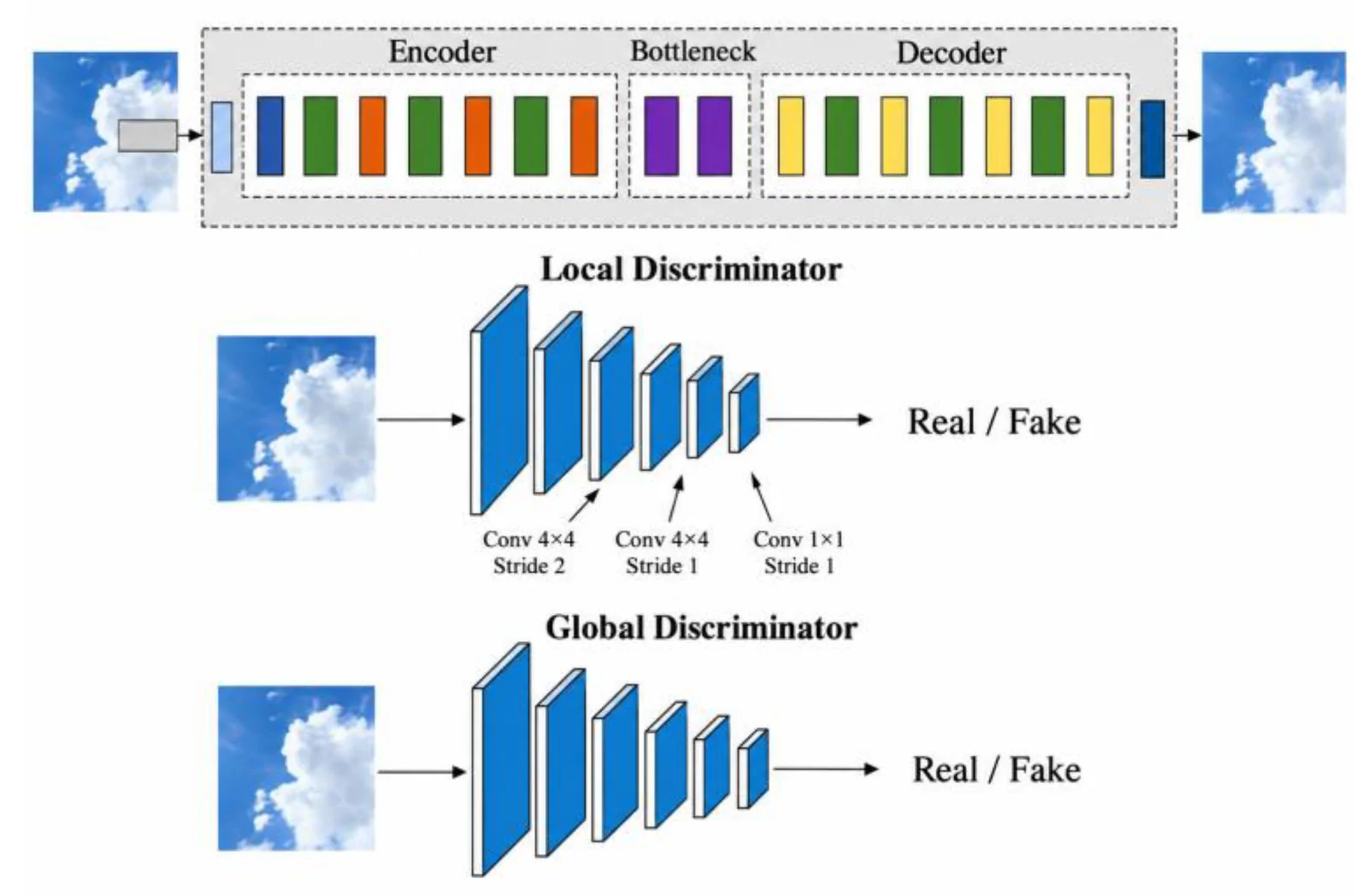
Network structure for ground-based cloud-image restoration.

**Figure 3:**
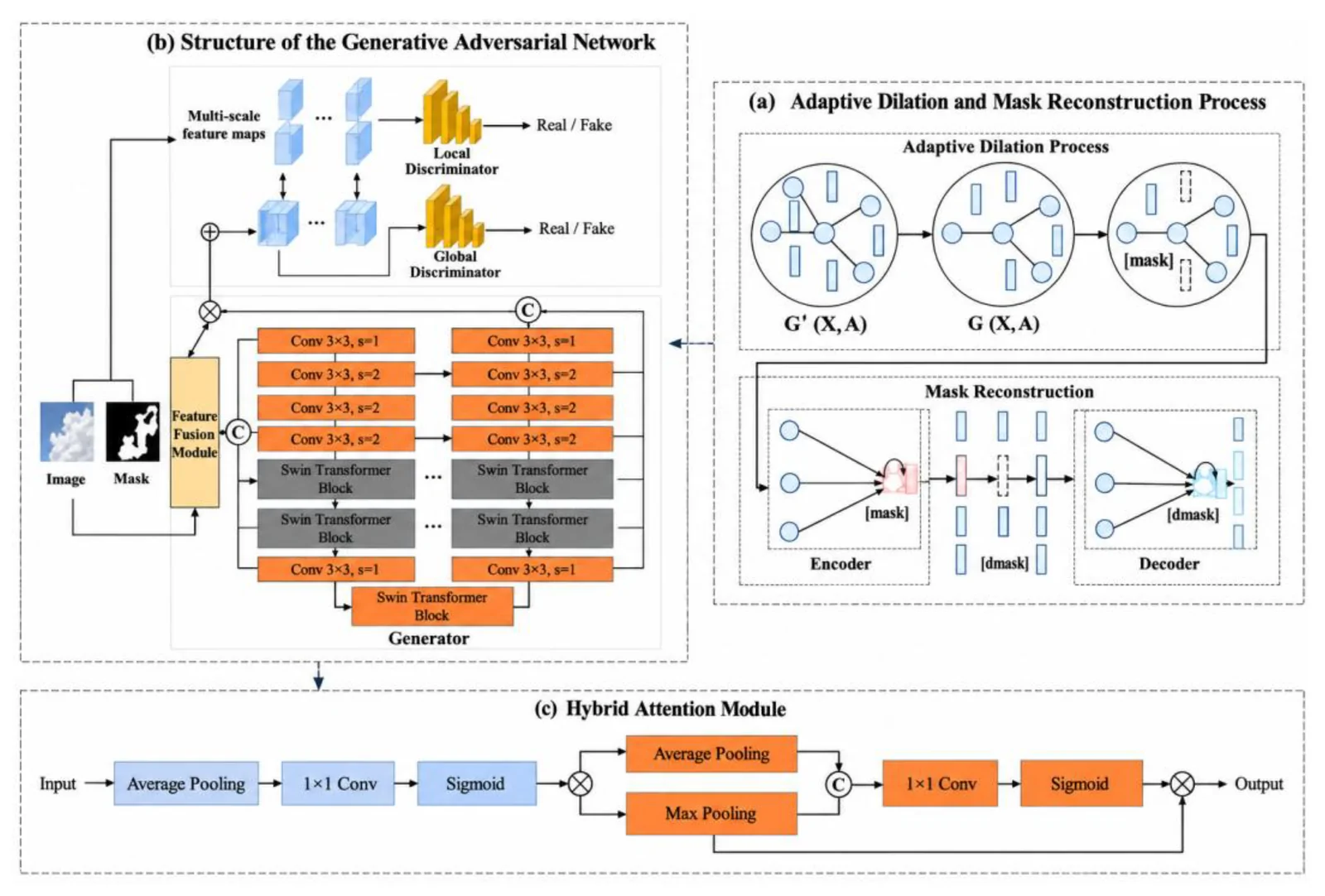
Adaptive cloud-image enhancement workflow and key module structures. Colors are used only to distinguish functional modules; geometric symbols denote the corresponding operations labeled in the figure, and arrows indicate data flow.

**Figure 4:**
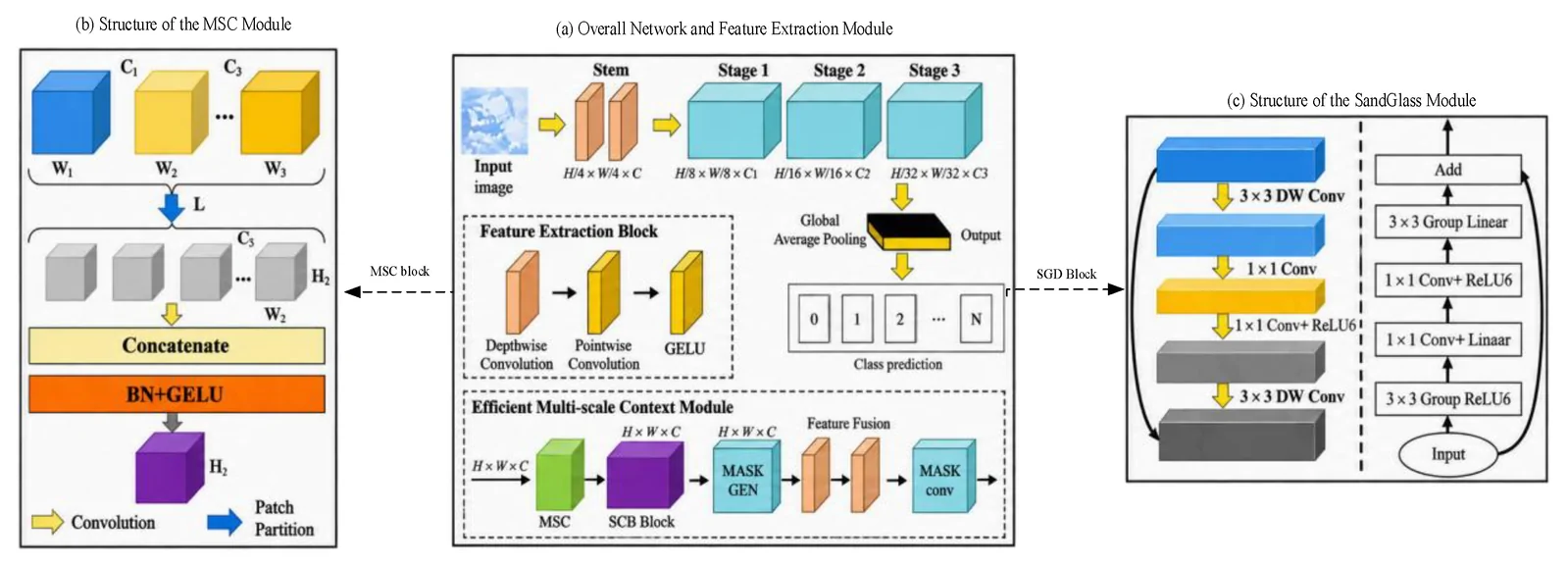
Improved CNN-Transformer network for cloud-image feature extraction.

**Figure 5:**
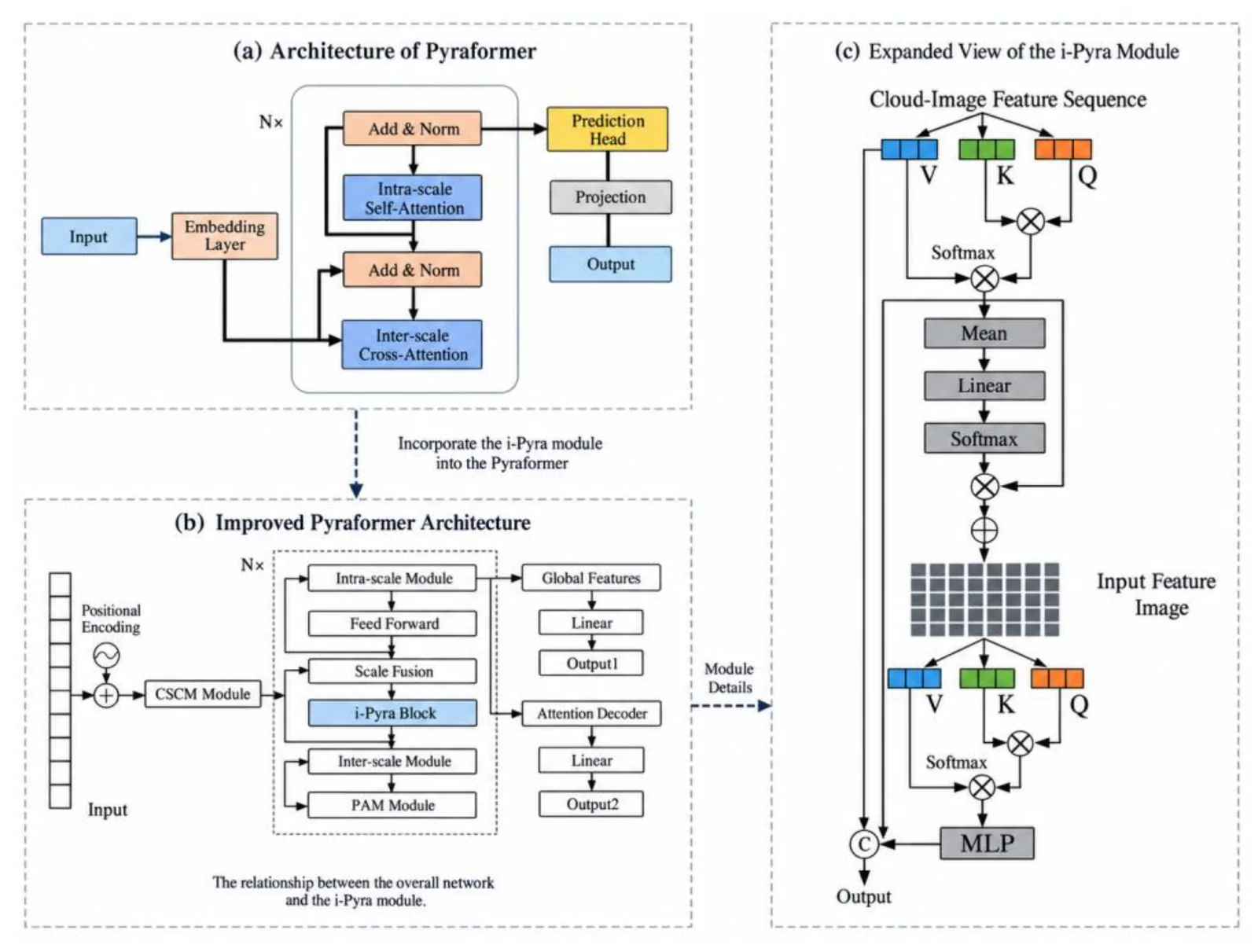
Basic Pyraformer architecture and modified forecasting model.

**Figure 6:**
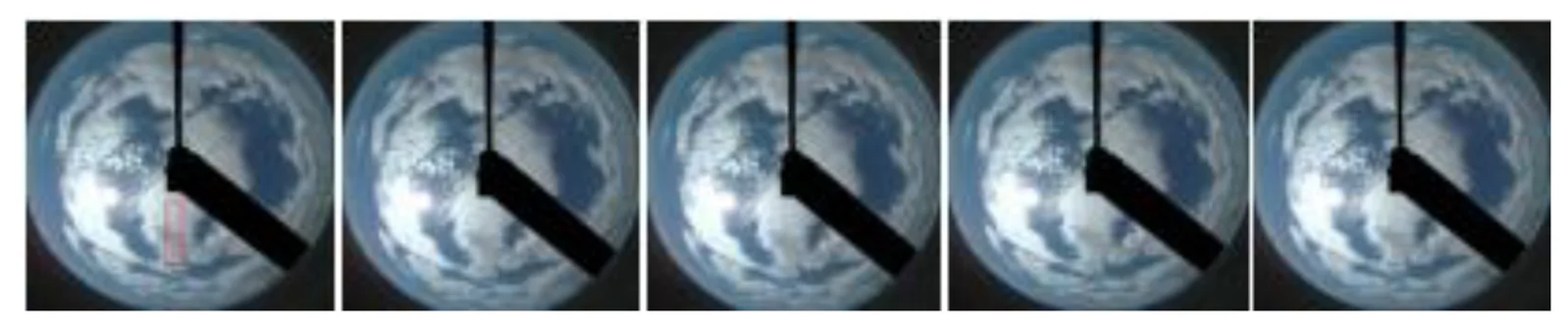
Comparison of cloud-image-restoration results.

**Figure 7:**
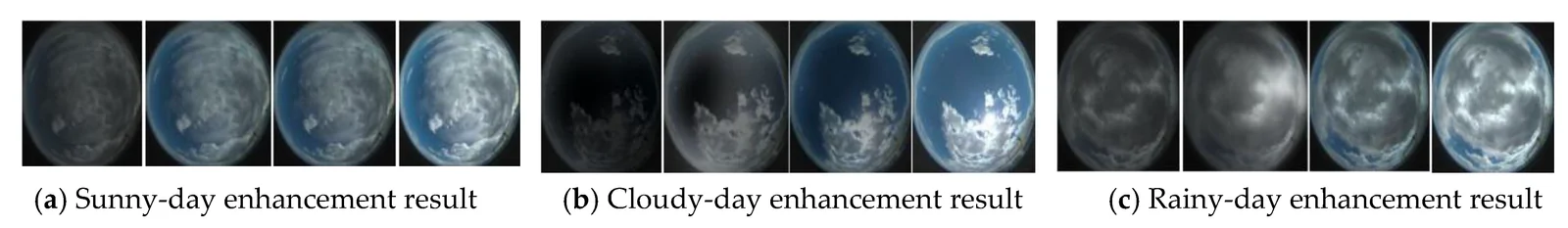
Comparison of cloud-image enhancement results obtained by different algorithms.

**Figure 8:**
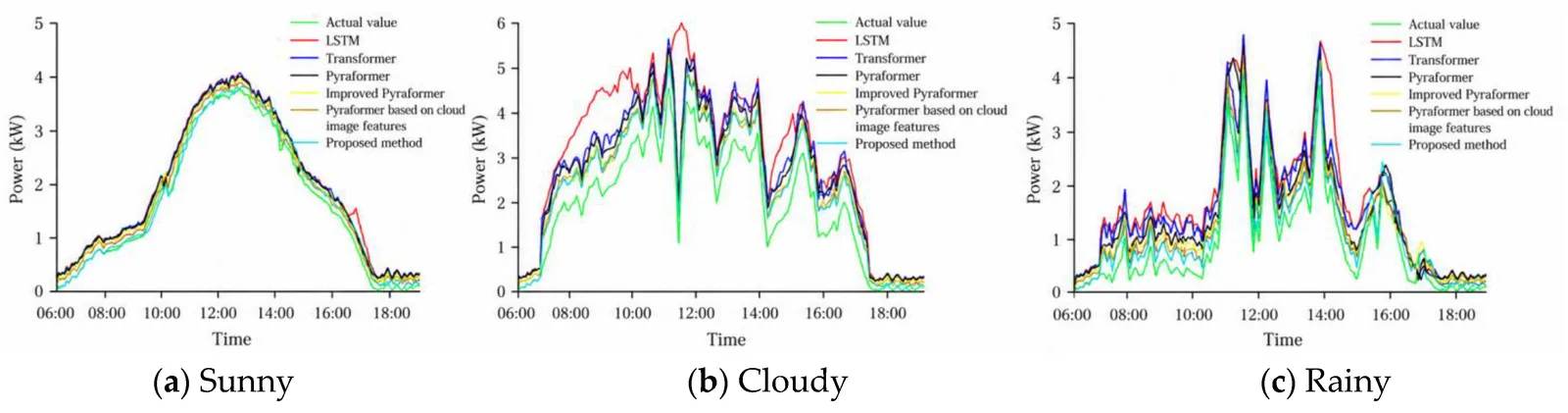
Comparison of predictions from different models on typical weather days.

**Table 1:** Comparison between existing methods and the proposed method.

Study	Main Input	Core Method	Research Focus
Xiang et al. [19]	PV power time series	TCN-ECANet-GRU hybrid model	Temporal feature modeling of power series
Hu et al. [30]	Thirteen features extracted from ground-based cloud images	Cloud identification, matching, and motion prediction	Cloud-image feature extraction and motion-information use
Hu et al. [32]	Ground-based cloud images and cloud dynamic features	Cloud-motion vector estimation and RBF neural network	Modeling of shading-cloud dynamic features
Wang et al. [33]	Ground-based cloud images and chaotic features	Cloud-image feature extraction and hybrid forecasting	Integrated use of cloud-image information and chaotic features
Proposed method	Ground-based cloud images and historical-power series	MAGAN enhancement, improved CNN-Transformer, LTF, and improved Pyraformer	Enhanced cloud-image representation, cross-modal interaction, and multi-scale temporal modeling

**Table 2:** Main parameter settings of the model and training process.

Parameter	Value
Number of Pyraformer pyramidal-attention layers	4
Number of attention heads	4
Number of adjacent nodes A	3
CSCM kernel size C	4
Convolution stride	4
Input sequence length L	16
Forecast steps H	4
Sampling and forecasting step Delta t	15 min
Cloud-image feature dimension $d_{c}$	128
Power feature dimension $d_{p}$	16
Fused-feature dimension $d_{h}$	128
Low-rank fusion rank R	8
i-Pyra window length w	8
Mask ratio	0.25
Batch size	64
Epoch	10
Optimizer	Adam
Learning rate	0.0001
Dropout rate	0.1
Random seed	2021

**Table 3:** Correlation coefficients between meteorological variables and PV power.

Feature Variable	Correlation Coefficient
Irradiance	0.963
Air temperature	0.489
Humidity	−0.436
Ground wind direction	0.142
Ground wind speed	−0.379
Cloud cover	0.791

**Table 4:** Quantitative comparison of cloud-image-restoration algorithms.

Method	PSNR	RMSE
DMFN	25.324	14.285
CTSDG	26.145	13.026
MFCL	26.264	12.956
Proposed method	26.852	11.463

**Table 5:** PSNR and SSIM of different cloud-image enhancement methods.

Method	PSNR/dB	SSIM
Original image	5.87	0.58
Standard Retinex	6.78	0.63
Bilateral-filter Retinex	6.97	0.69
Proposed method	7.25	0.75

**Table 6:** Prediction errors of each model on typical weather days.

W	Metric	LSTM	Transformer	Pyraformer	Improved Pyraformer	Cloud-Fusion Pyraformer	Proposed Method
Sun	MAE/%	22.8	21.4	18.2	26.2	15.6	12.4
	MAPE/%	30.5	29.1	26.3	24.5	15.8	12.7
	RMSE/%	25.7	24.6	21.2	24.6	18.4	15.6
Cloudy	MAE/%	42.6	40.3	35.2	39.7	38.5	35.2
	MAPE/%	40.1	38.7	36.5	21.8	18.5	16.7
	RMSE/%	62.5	60.8	56.1	59.4	58.6	51.6
Rain	MAE/%	41.2	39.8	37.5	26.3	25.7	23.1
	MAPE/%	44.6	43.2	41.3	36.5	31.2	28.9
	RMSE/%	50.4	48.9	46.3	45.6	43.1	38.7

**Table 7:** Prediction errors on the independent test set.

Model	MAE/%	MAPE/%	RMSE/%	MAE Standard Deviation/%
LSTM	34.2	36.5	44.1	7.8
Transformer	32.6	35.2	42.7	7.5
Pyraformer	28.9	33.1	39.8	6.9
Improved Pyraformer	27.4	26.3	38.9	6.6
Cloud-feature and sample-weighted Pyraformer	25.1	20.9	38.2	6.2
Proposed method	21.8	17.6	32.4	5.4

**Table 8:** Results of the ablation experiments. Here, √ indicates that a module is retained, whereas × indicates that it is removed.

Adaptive Enhancement	Mask Reconstruction	Mixed Attention	Swin Transformer	SSIM	MAE/%	MAPE/%	RMSE/%
×	√	√	√	0.67	26.6	22.6	39.7
√	×	√	√	0.72	25.3	20.9	36.1
√	√	×	√	0.68	26.1	21.6	39.4
√	√	√	×	0.71	25.7	20.9	37.3
√	√	√	√	0.75	23.6	19.4	35.3

**Table 9:** Relationship between fusion-layer parameter count and rank.

Fusion Method	Parameter Count	Relative to Full Bilinear Fusion	Parameter-Reduction Condition in Equation (17)
Full bilinear fusion	280,832	1.000	—
Low-rank fusion R = 2	37,504	0.134	R < 15.0, satisfied
Low-rank fusion R = 4	74,880	0.267	R < 15.0, satisfied
Low-rank fusion R = 8	149,632	0.533	R < 15.0, satisfied
Low-rank fusion R = 16	299,392	1.066	R > 15.0, not satisfied

**Table 10:** Comparison of pairwise attention computation.

Attention Form	Pairwise Computation	Value When N = 16	Relative to Global Attention
Global self-attention	O(*N*^2^*d_k_*)	256	1.000
Pyramidal attention PAM	O(*NAd_k_*)	48	0.188
Window attention (i-Pyra, Equation (19))	O(*Nwd_k_*)	128	0.500

## Data Availability

The original contributions presented in the study are included in the article and further inquiries can be directed to the corresponding author.
